# Supplementary material for: Two Rac1 pools integrate the direction and coordination of collective cell migration
Source: Nat Commun. 2022 Oct 12;13:6014. doi: 10.1038/s41467-022-33727-6 (PMC9556596; doi:10.1038/s41467-022-33727-6)
Supplement: Supplementary file 1 — Supplementary Information [file 41467_2022_33727_MOESM1_ESM.pdf]

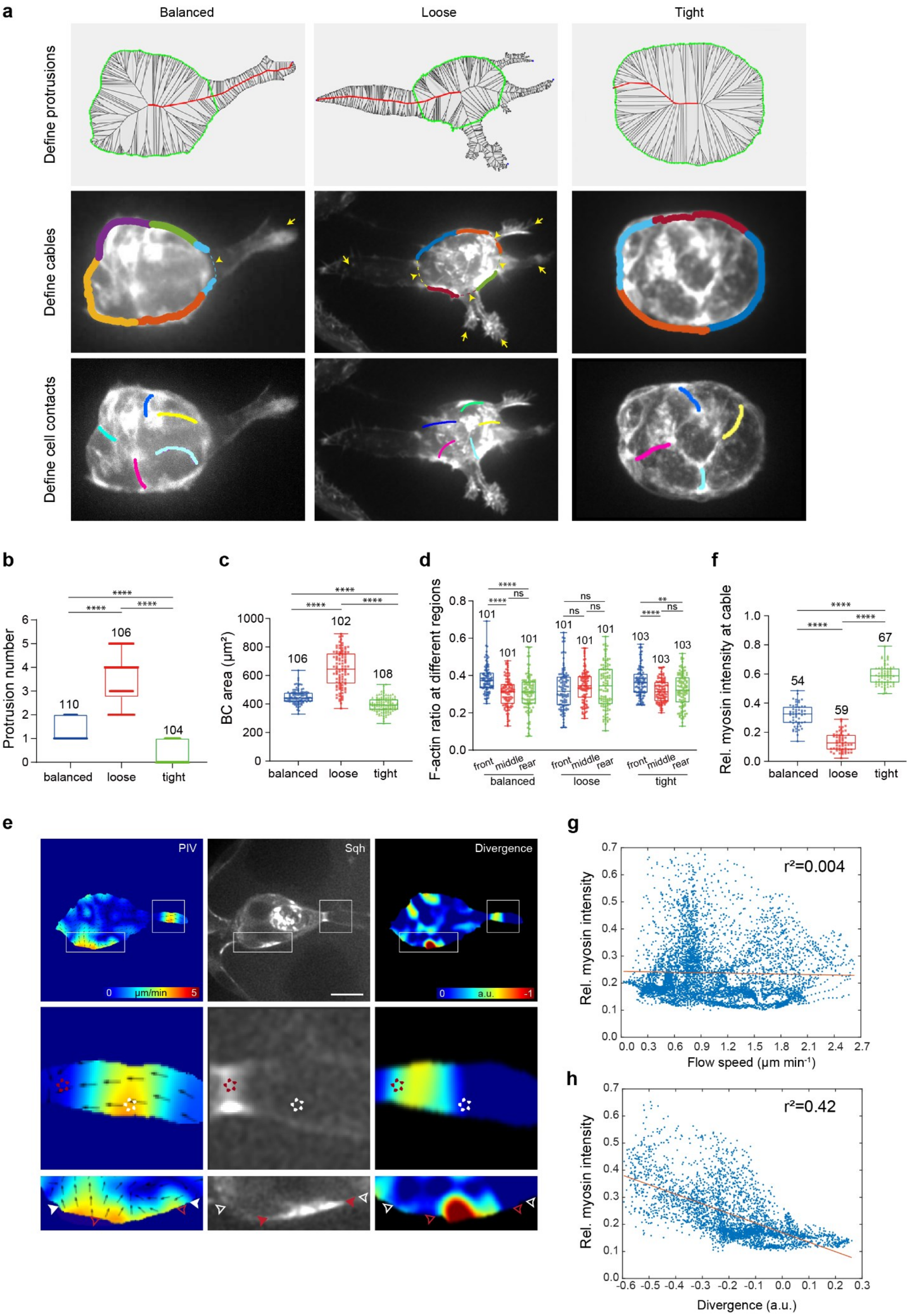

a
Balanced
Loose
Tight
Define protrusions
Define cables
Define cell contacts
b
Protrusion number
110
106
104
balanced
loose
tight
c
BC area (μm²)
106
102
108
balanced
loose
tight
d
F-actin ratio at different regions
front
middle
rear
balanced
loose
tight
ns
f
Rel. myosin intensity at cable
54
59
67
balanced
loose
tight
e
PIV
Sqh
Divergence
μm/min
a.u.
g
Rel. myosin intensity
r²=0.004
Flow speed (μm min⁻¹)
h
Rel. myosin intensity
r²=0.42
Divergence (a.u.)

Supplementary Figure 1. Analyses of subcellular F-actin signals and actin flows in migrating border cell groups.

a. Definition of protrusions, cables and the cell-to-cell contacts in representative balanced, loose or tight border cell groups expressing LifeAct-GFP. Different colors marking either cables in one border cells or cell-cell contacts between two border cells. Yellow arrows marking protrusions, yellow arrowheads marking broken cables. **b, c.** Quantifications of protrusion number (**b**) and border cell area (**c**) in the indicated border cell groups. **d.** Ratio quantification of total peripheral F-actin signals distributed at the front, middle or rear cells in the indicated border cell groups. **e.** Representative PIV and divergence analyses of actin flows in balanced border cell group expressing LifeAct-GFP and Sqh-mCherry for F-actin and Myosin-II signals. PIV analysis performed on the LifeAct-GFP signals to highlight the direction and magnitude of actin flows. Divergence calculated from the actin flowfield to highlight the region where actin flows from different directions converge and thus Myosin-II signals accumulate at cables or protrusion-cable boundaries. a.u. means arbitrary unit for divergence level. White arrowheads (cables) or stars (protrusions) marking high PIV regions, while red arrowheads (cables) or stars (protrusions) marking negative divergence regions. **f.** Quantification of relative Myosin-II signals accumulated at cables in the indicated border cell groups. **g, h.** Scatter plot comparing a random sample of points in the actin flowfield for relative actin flow speed and relative Myosin-II intensity (**g**), or for divergence and relative Myosin-II intensity (**h**). Note the positive relationship between negative divergence and relative Myosin-II intensity (n = 6156 random points, 9 biologically independent samples); but no significant relationship between actin flow speed and relative Myosin-II intensity (n = 5876 random points, 9 biologically independent samples). Scale bars are 10 µm in **e**. Boxplot shows medians, 25th and 75th percentiles as box limits, minimum and maximum values as whiskers; each datapoint is displayed as a dot (from n biologically independent samples for each border cell group), in **b, c, d** and **f**. P values by two-sided Mann–Whitney test have been listed in Supplementary Note 1. Source data are provided as a Source Data file.

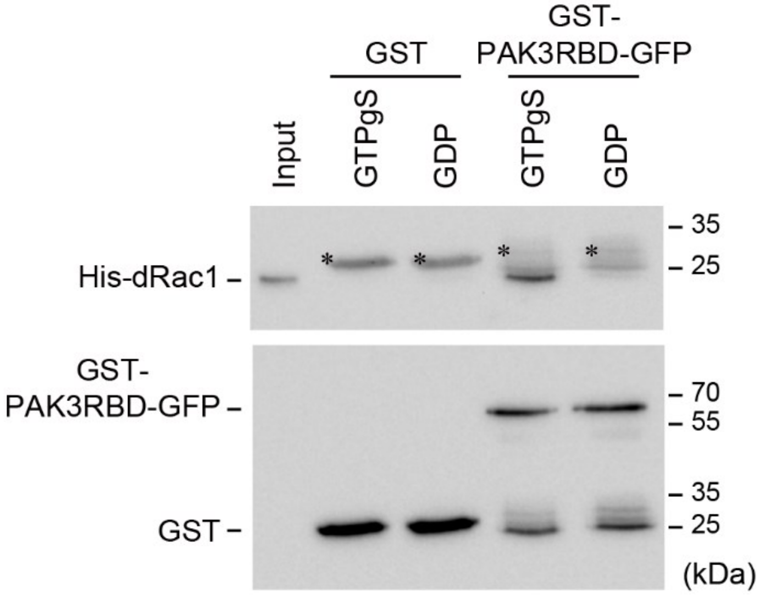

Supplementary Figure 2. Control experiments for the binding specificity of GTP-loaded dRac1 to PAK3RBD-GFP.

GTPγS- or GDP-loaded His-dRac1 was incubated with GST or GST-PAK3RBD-GFP recombinant proteins purified from *E. coli*. Input and pull-down samples were analysed by immunoblotting with antibodies against His (top panel) or GST (bottom panel). Asterisk marks non-specific signals. The results have been successfully repeated from the at least 3 independent experiments.

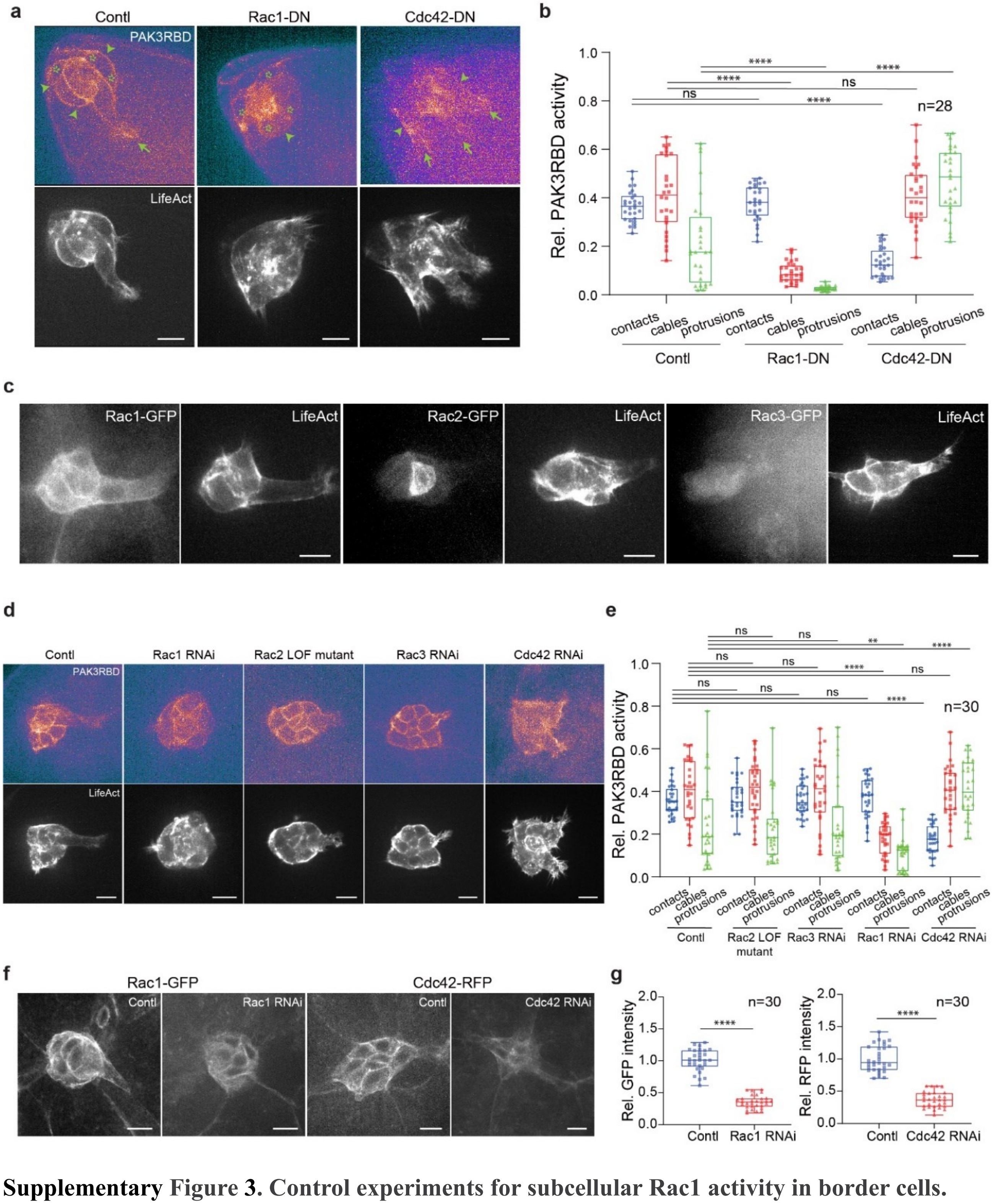

Supplementary Figure 3. Control experiments for subcellular Rac1 activity in border cells.
a. Representative PAK3RBD-GFP and F-actin images in border cell groups expressing Rac1DN, Cdc42DN and control (yw; named as WT), together with LifeAct-RFP to discriminate and label different regions enriched with subcellular F-actin. Since the expression of Rac1DN and Cdc42DN in border cells led to the defect in border cell detachment, we chose all these three border cell

groups during detachment for the comparison. Green arrows marking protrusions, green arrowheads marking cables, while green stars marking border cell-cell contacts. **b.** Quantification of relative PAK3RBD-GFP intensity located at contacts, cables or protrusions in the indicated border cell groups. **c.** Representative GFP and F-actin images in border cell groups expressing Rac1-GFP, Rac2-GFP and Rac3-GFP (endogenous patterns), together with LifeAct-RFP to monitor subcellular F-actin signals. Rac1-GFP vs. Rac2-GFP in border cells (but not two internal polar cells): 1+/-0.168 vs. 0.24+/-0.096, and Rac1-GFP vs. Rac3-GFP in border cells: 1+/-0.168 vs. 0.17+/-0.071, from n=35 independent samples. **d.** Representative PAK3RBD-GFP and F-actin images in border cell groups expressing Rac1 RNAi, Rac3 RNAi or Cdc42 RNAi, or with Rac2 LOF mutant, together with LifeAct-RFP to discriminate different regions enriched with subcellular F-actin. **e.** Quantification of relative PAK3RBD-GFP intensity located at contacts, cables or protrusions in the indicated border cell groups. **f.** Representative GFP and RFP images in border cell groups expressing Rac1-GFP (and Rac1 RNAi or control), or Cdc42-RFP (and Cdc42 RNAi or control), from fixed imaging. **g.** Quantification of relative GFP/RFP intensity in total border cell groups in the indicated border cell groups. Scale bars are 10 μm in **a, c, d, f**. Boxplot shows medians, 25th and 75th percentiles as box limits, minimum and maximum values as whiskers; each datapoint is displayed as a dot (from n biologically independent samples for each border cell group), in **b, e, g**. P values by two-sided Mann–Whitney test have been listed in Supplementary Note 1. Source data are provided as a Source Data file.

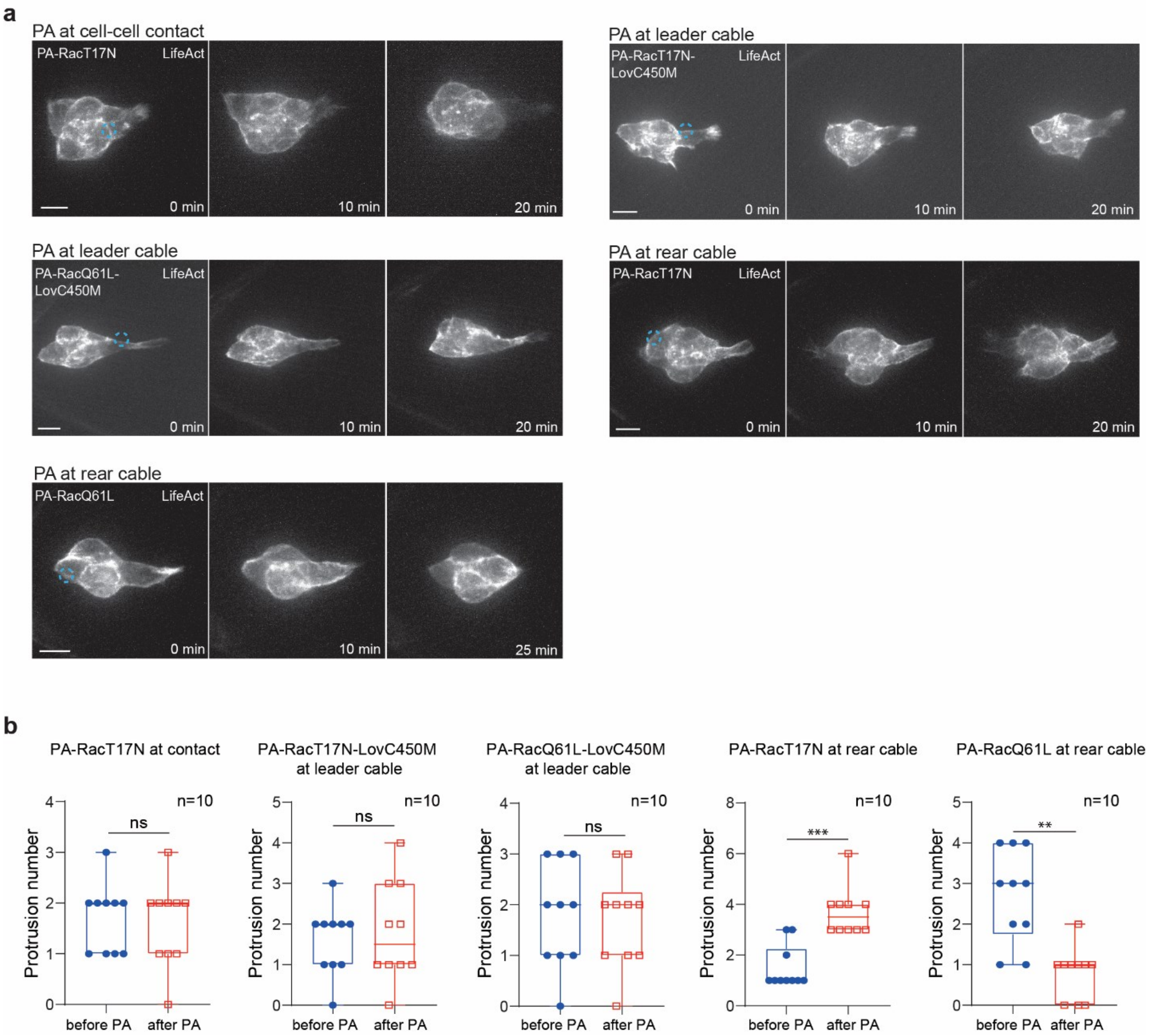

Supplementary Figure 4. Control experiments for PA-Rac effects.

a. Representative time-lapse images of border cell groups expressing the indicated PA-Rac forms, together with LifeAct-RFP to monitor subcellular F-actin signals. Dotted blue circle labelling the PA regions with blue light illumination, either at cables in leader or rear border cells, or at border cell-to-cell contacts. PA means photo-activation. **b**. Quantification of protrusion number before and after 18-25–minute photo-activation of the indicated PA-Rac forms at cables or border cell-to-cell contacts. Scale bars are 10 μm in **a**. Boxplot shows medians, 25th and 75th percentiles as box limits, minimum and maximum values as whiskers; each datapoint is displayed as a dot (from n biologically independent samples for each border cell group) in **b**. P values by two-sided Mann–Whitney test have been listed in Supplementary Note 1. Source data are provided as a Source Data file.

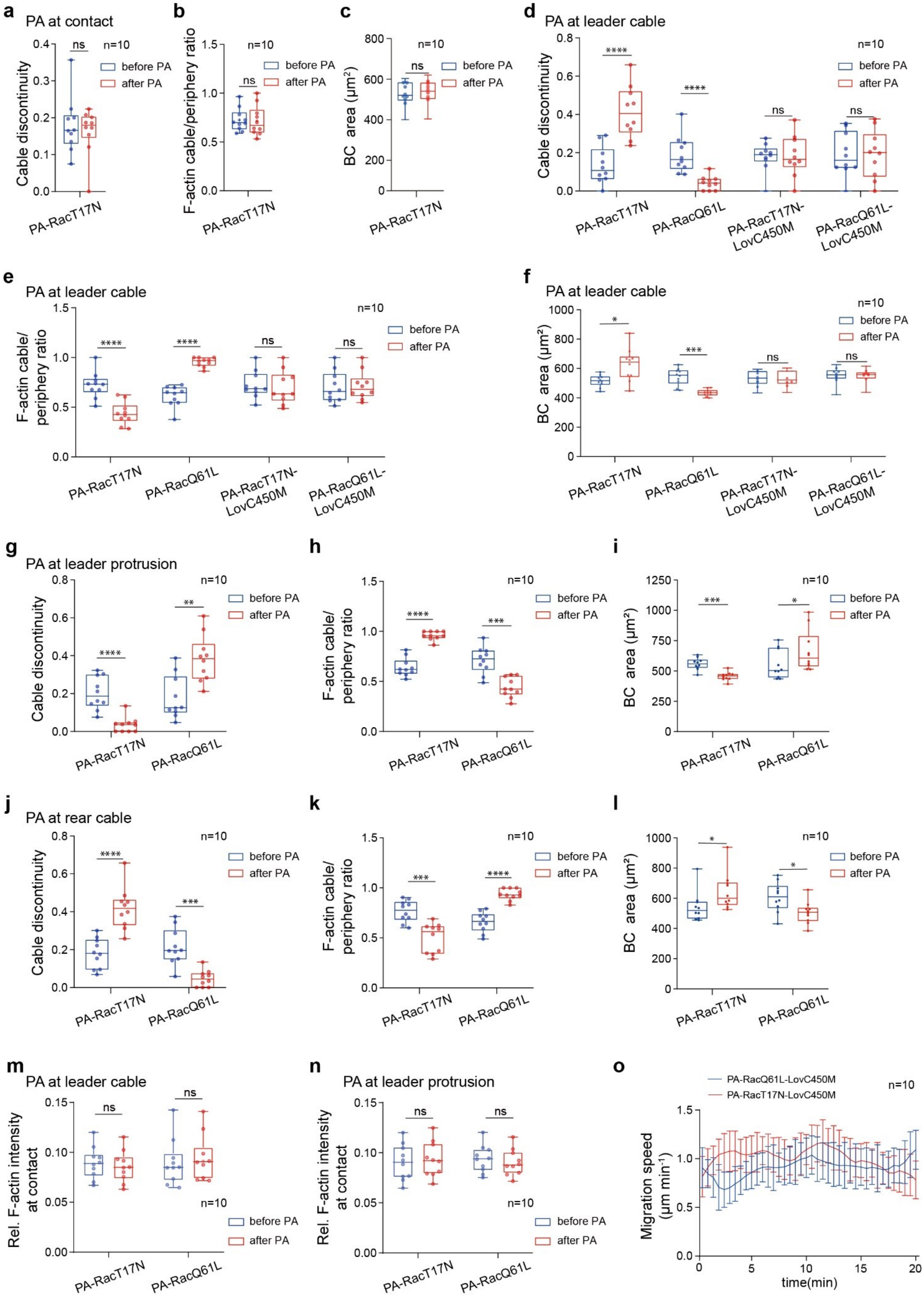

a
PA at contact
n=10
ns
before PA
after PA
Cable discontinuity
PA-RacT17N
b
F-actin cable/periphery ratio
PA-RacT17N
c
BC area (μm²)
PA-RacT17N
d
PA at leader cable
PA-RacT17N
PA-RacQ61L
PA-RacT17N-LovC450M
PA-RacQ61L-LovC450M
e
PA at leader cable
F-actin cable/periphery ratio
f
PA at leader cable
BC area (μm²)
g
PA at leader protrusion
Cable discontinuity
h
F-actin cable/periphery ratio
i
BC area (μm²)
j
PA at rear cable
Cable discontinuity
k
F-actin cable/periphery ratio
l
BC area (μm²)
m
PA at leader cable
Rel. F-actin intensity at contact
n
PA at leader protrusion
Rel. F-actin intensity at contact
o
PA-RacQ61L-LovC450M
PA-RacT17N-LovC450M
Migration speed (μm min⁻¹)
time(min)

Supplementary Figure 5. Quantifications of the PA-Rac effect.
a-c, d-f, g-i, j-l. Quantifications of cable discontinuity (**a, d, g, j**), the ratio between cable F-actin signals and total peripheral F-actin signals (**b, e, h, k**), and total cell area (**c, f, i, l**) in the indicated border cell groups, before and after 18-25−minute photo-activation at the indicated regions. **m, n.** Quantifications of relative F-actin intensity located at the border cell-to-cell contacts in the border cell groups expressing either PA-RacT17N or PA-RacQ61L, before and after 18-25−minute photo-activation of PA-Rac at leader border cell cables (**m**) or protrusions (**n**). **o.** Time-lapse quantifications of mean migration speed (μm per minute) in the border cell groups expressing either PA-RacQ61L-LovC450M or PA-RacT17-LovC450M, before and after focal illumination of PA-Rac-LovC450M at leader border cell cables. Boxplot shows medians, 25th and 75th percentiles as box limits, minimum and maximum values as whiskers; each datapoint is displayed as a dot (from n biologically independent samples for each border cell group), in **a-n**. Data are presented as mean values +/− SD in **o** (from n biologically independent samples for each border cell group). P values by two-sided Mann–Whitney test have been listed in Supplementary Note 1. Source data are provided as a Source Data file.

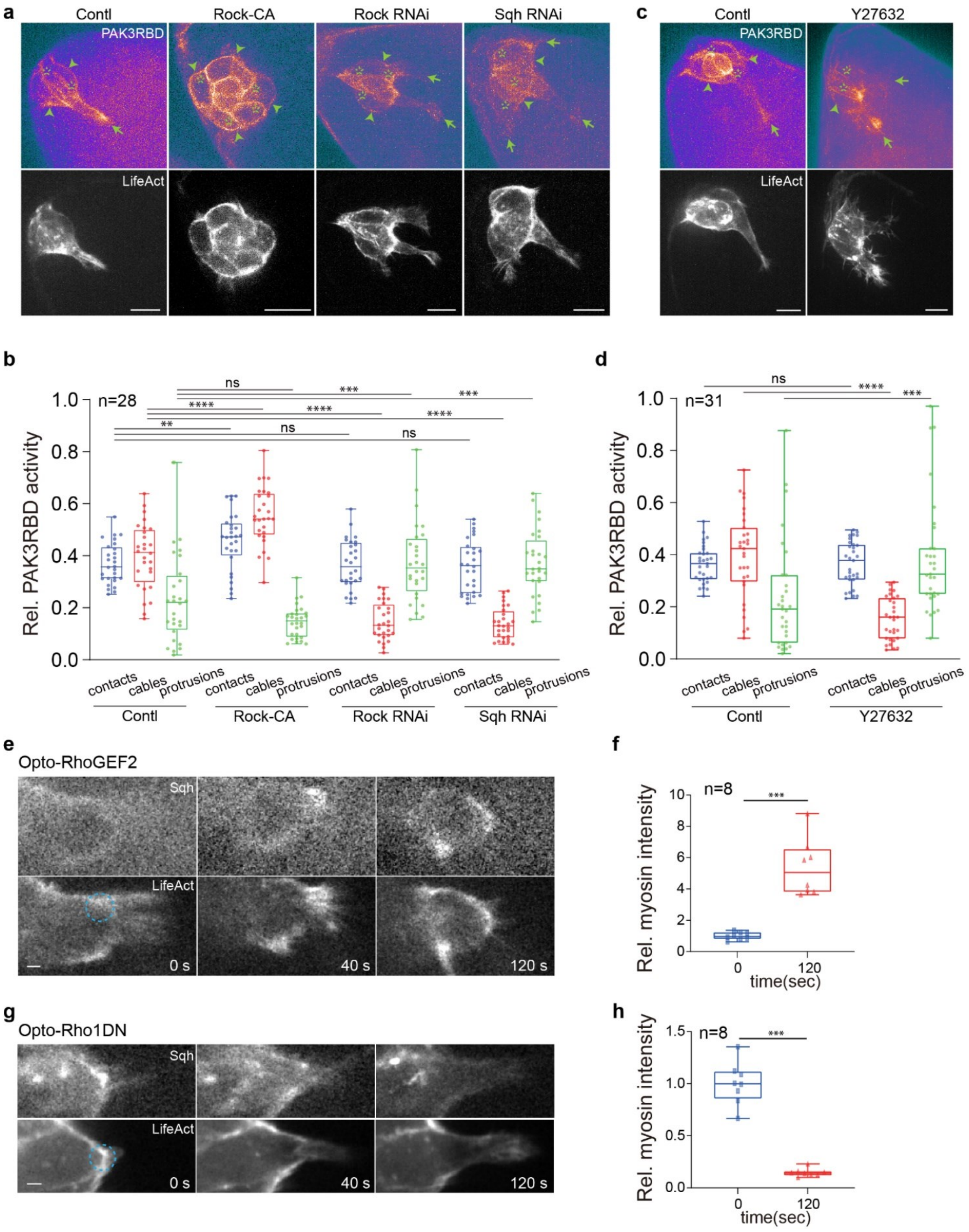
a
Contl
Rock-CA
Rock RNAi
Sqh RNAi
PAK3RBD
LifeAct
c
Contl
Y27632
PAK3RBD
LifeAct
b
n=28
ns
***
****
**
Rel. PAK3RBD activity
contacts
cables
protrusions
Contl
Rock-CA
Rock RNAi
Sqh RNAi
d
n=31
Rel. PAK3RBD activity
Contl
Y27632
e
Opto-RhoGEF2
Sqh
LifeAct
0 s
40 s
120 s
f
n=8
Rel. myosin intensity
time(sec)
g
Opto-Rho1DN
Sqh
LifeAct
0 s
40 s
120 s
h
n=8
Rel. myosin intensity
time(sec)

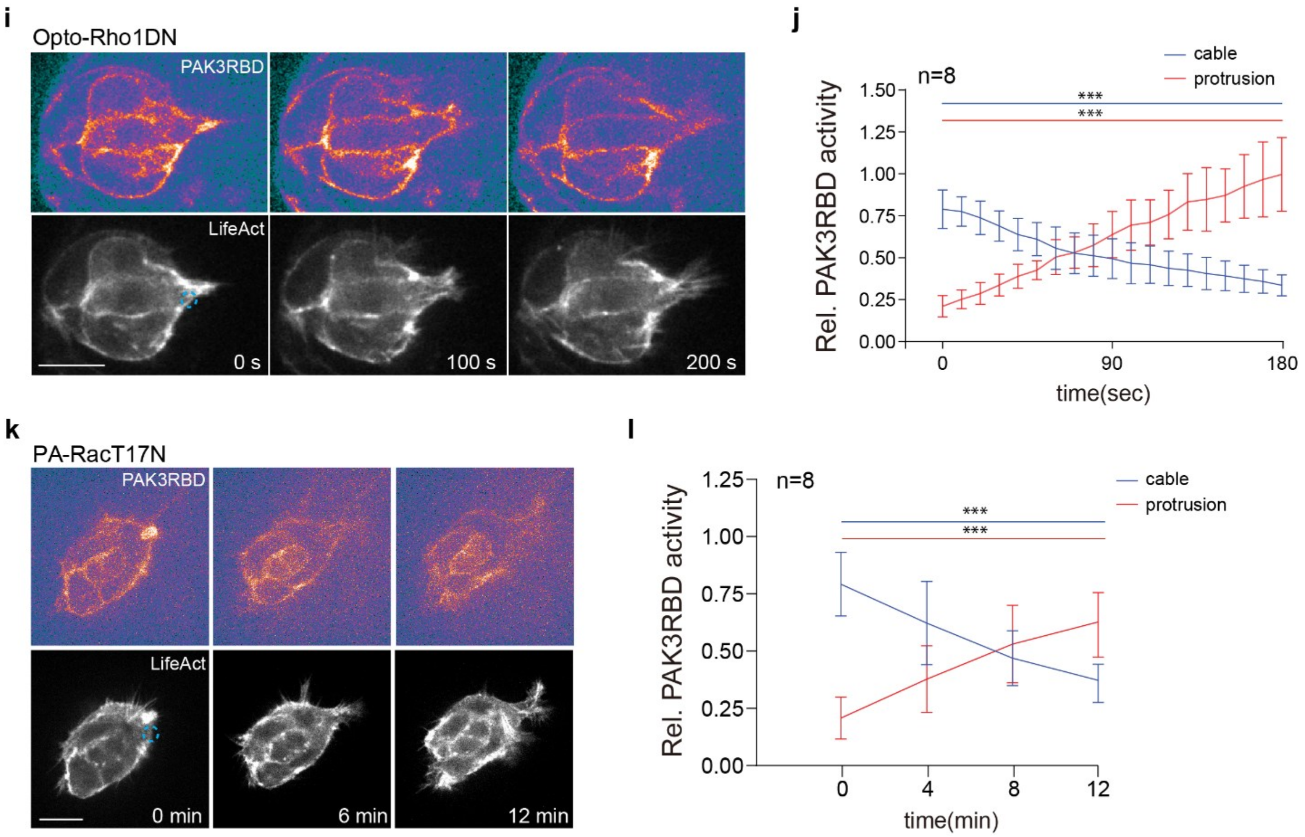

Supplementary Figure 6. Rho1 signalling governs cable Rac1 activity to support supracellular cables.

a, c. Representative PAK3RBD-GFP and F-actin images in border cell groups with the expression of Rock-CA, Rock RNAi, Myosin-II RNAi or control (**a**), and with the treatment of Y27632 or DMSO control (**c**), together with LifeAct-RFP for different subcellular F-actin signals. Green arrows marking protrusions, green arrowheads marking cables, while green stars marking border cell-cell contacts. **b, d.** Quantification of relative PAK3RBD-GFP intensity located at contacts, cables or protrusions in border cell groups with the indicated genetic backgrounds (**b**) and treatment (**d**). **e, g.** Representative time-lapse Sqh-GFP and F-actin images in border cell groups expressing Opto-RhoGEF2 and LifeAct-RFP (**e**) and Opto-Rho1DN and LifeAct-RFP (**g**), before and after blue light illumination at the cable regions near border cell leading protrusions. Dotted blue circle labelling the PA regions with blue light illumination. PA means photo-activation. **f, h.** Quantification of relative Myosin-II intensity before and after 120-second photo-activation of Opto-RhoGEF2 (**f**) and Opto-Rho1DN (**h**) at the cable regions near leading protrusions. **i, k.** Representative time-lapse PAK3RBD-GFP and F-actin images in border cell groups expressing Opto-Rho1DN and LifeAct-RFP (**i**) and PA-RacT17N and LifeAct-RFP (**k**), before and after blue light illumination at the cable regions of one border cell. **j, l.** Time-lapse quantification of relative PAK3RBD-GFP intensity located at cables or protrusions in one cell with Opto-Rho1DN photo-treatment (**j**) and in the whole groups with PA-RacT17N photo-treatment at one border cell cable (**l**), before and after photo-activation. Scale bars are 10 μm in **a, c, i** and **k**, 2 μm in **e** and **g**. Boxplot shows medians, 25th and 75th percentiles as box limits, minimum and maximum values as whiskers; each datapoint is displayed as a dot (from n biologically independent samples for each border cell group), in **b, d, f and h.** Data are presented as mean values +/− SD in **j** and **l** (from n

biologically independent samples for each border cell group). P values by two-sided Mann–Whitney test have been listed in Supplementary Note 1. Source data are provided as a Source Data file.

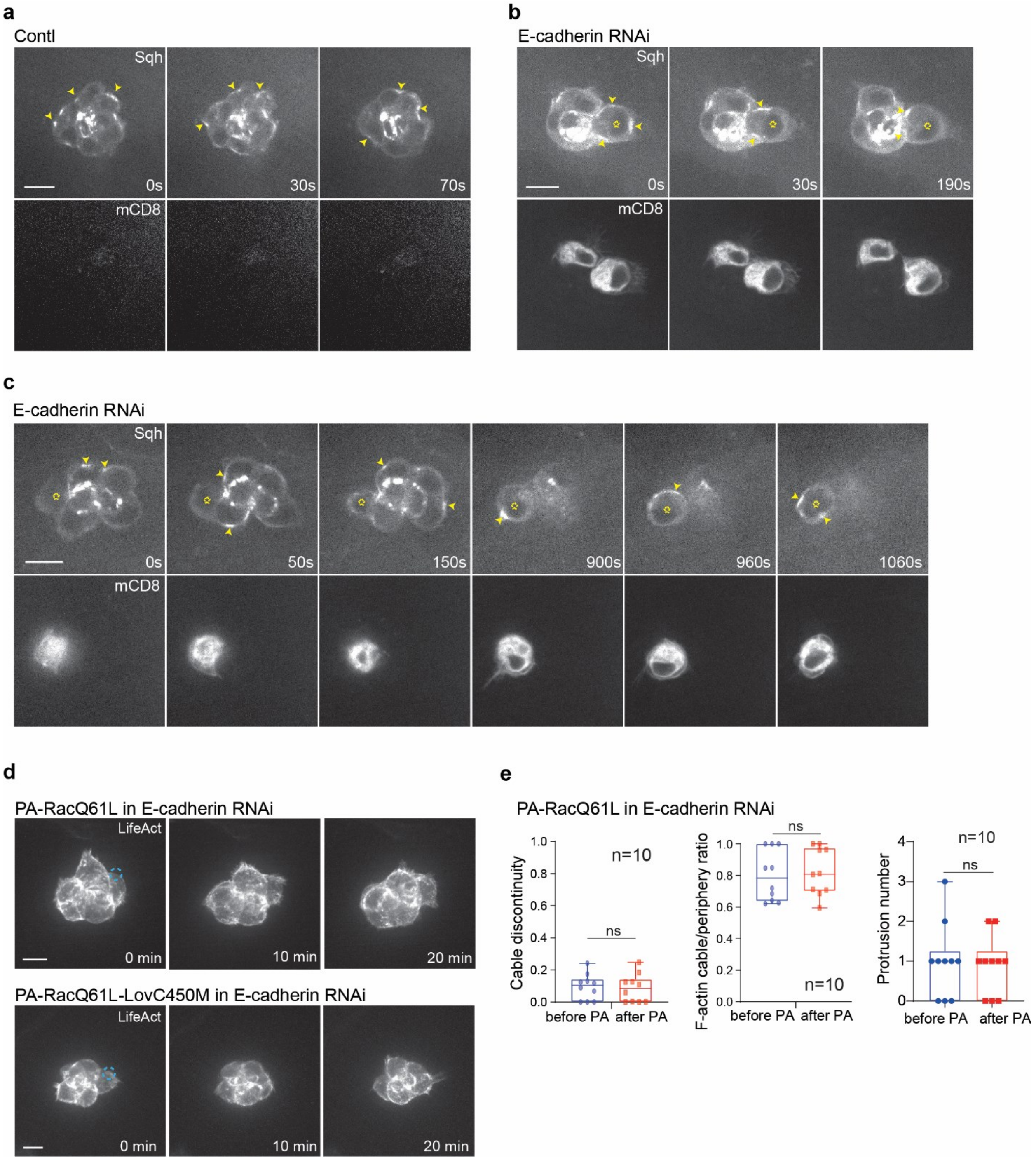

Supplementary Figure 7. Confirmation for the role of E-cadherin on controlling pulsed Myosin-II signal transmission at supracellular cables.

a. Time-lapse images of Myosin-II flows in one representative WT border cell group expressing Sqh-GFP but not mCD8-RFP signals. Yellow arrowheads marking Myosin-II pulsed flows. **b.** Time-lapse images of Myosin-II flows in one representative Sqh-GFP expressing border cell group, in which 1-2 border cell clone(s) express E-cadherin RNAi (Shg^{RNAi}) together with mCD8-RFP as the clone marker. Yellow arrowheads marking Myosin-II pulsed flows, while yellow stars marking

the clonal cell. **c.** Time-lapse images of Myosin-II flows in one representative Sqh-GFP expressing border cell group, in which 1-2 border cell clone(s) express E-cadherin RNAi (Shg^{RNAi}) together with mCD8-RFP as the clone marker. Yellow arrowheads marking Myosin-II pulsed flows, while yellow stars marking the clonal cell. The plane of time-lapse images between 900 seconds and 1060 seconds is 5 μm higher than the one between 0 seconds and 150 seconds. The results (**a-c**) have been successfully repeated from the at least 4 independent experiments. **d.** Representative time-lapse F-actin images of border cell groups expressing PA-RacQ61L and E-cadherin RNAi (upper), or PA-RacQ61L-LovC450M and E-cadherin RNAi (lower), together with LifeAct-RFP to monitor subcellular F-actin signals, before and after photo-activation of PA-RacQ61L at one border cell. Dotted blue circle labelling the PA regions with blue light illumination. PA means photo-activation. **e.** Quantifications of cable discontinuity, the ratio between cable F-actin signals and total peripheral F-actin signals, and protrusion number in the border cell groups expressing PA-RacQ61L and E-cadherin RNAi, before and after 20–minute photo-activation at one border cell. Scale bars are 10 μm in **a-d**. Boxplot shows medians, 25th and 75th percentiles as box limits, minimum and maximum values as whiskers; each datapoint is displayed as a dot (from n biologically independent samples for each border cell group) in **e.** P values by two-sided Mann–Whitney test have been listed in Supplementary Note 1. Source data are provided as a Source Data file.

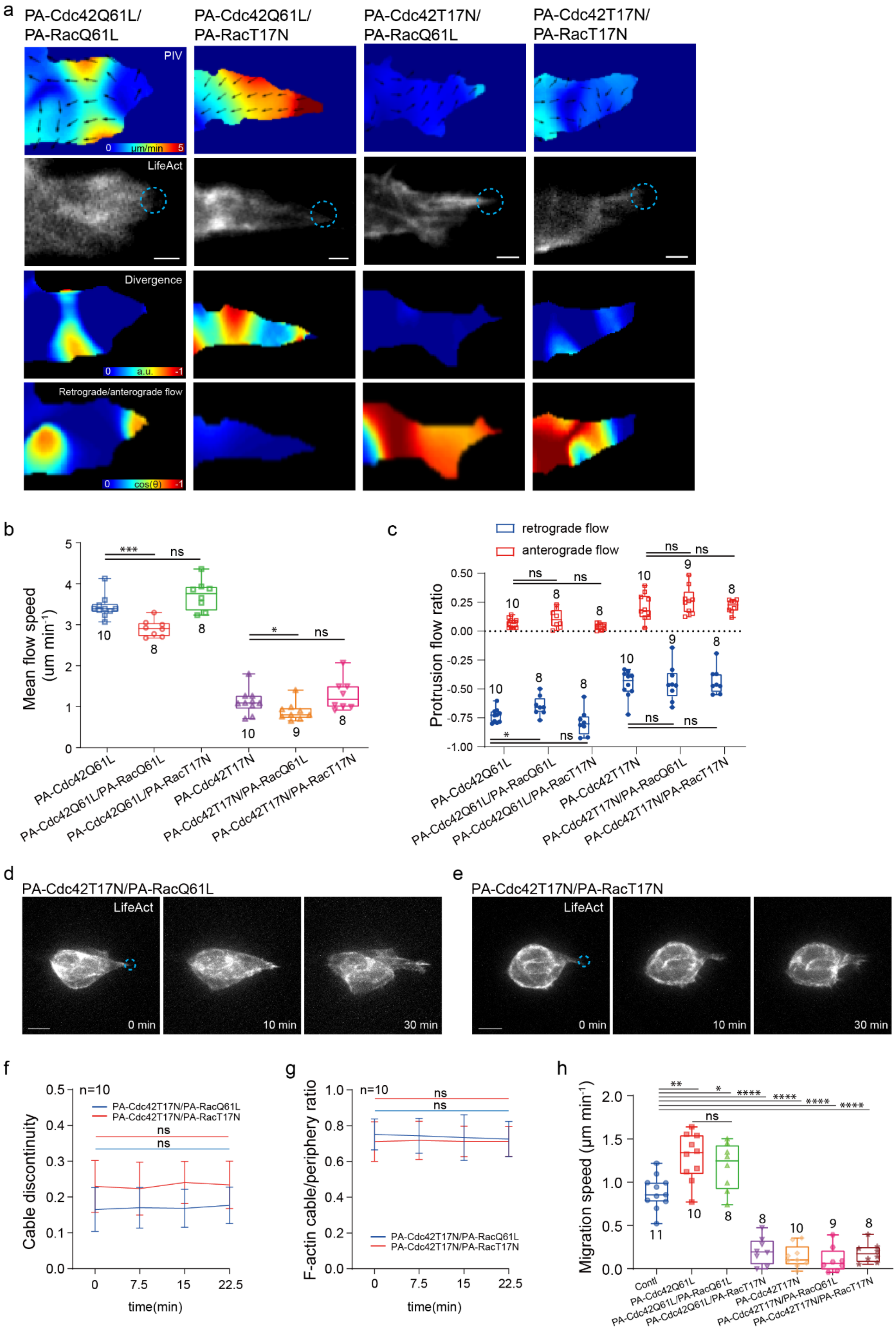

a
PA-Cdc42Q61L/
PA-RacQ61L
PA-Cdc42Q61L/
PA-RacT17N
PA-Cdc42T17N/
PA-RacQ61L
PA-Cdc42T17N/
PA-RacT17N
PIV
0 μm/min 5
LifeAct
Divergence
0 a.u. -1
Retrograde/anterograde flow
0 cos(θ) -1
b
Mean flow speed (um min⁻¹)
PA-Cdc42Q61L
PA-Cdc42Q61L/PA-RacQ61L
PA-Cdc42Q61L/PA-RacT17N
PA-Cdc42T17N
PA-Cdc42T17N/PA-RacQ61L
PA-Cdc42T17N/PA-RacT17N
c
retrograde flow
anterograde flow
Protrusion flow ratio
d
PA-Cdc42T17N/PA-RacQ61L
LifeAct
0 min
10 min
30 min
e
PA-Cdc42T17N/PA-RacT17N
f
Cable discontinuity
n=10
time(min)
g
F-actin cable/periphery ratio
h
Migration speed (μm min⁻¹)
Contl

Supplementary Figure 8. Confirmation for the role of Cdc42 on governing actin flows at protrusions and F-actin signal exchange between protrusion and cables for efficient border cell migration.

a. Representative PIV, divergence and retrograde/anterograde direction analyses of actin flows at leading protrusions in the border cell groups expressing PA-Cdc42Q61L/PA-RacQ61L, PA-Cdc42Q61L/PA-RacT17N, PA-Cdc42T17N/PA-RacQ61L or PA-Cdc42T17N/PA-RacT17N, together with LifeAct-GFP for F-actin signals. a.u. means arbitrary unit for divergence level. Dotted blue circle labelling the PA regions with blue light illumination. PA means photo-activation. **b.** Quantification of mean flow speed (μm per minute) at leading protrusions in the indicated border cell groups. **c.** Quantification of the occurrence ratio of retrograde and anterograde actin flows at leading protrusions in the indicated border cell groups. **d, e.** Representative time-lapse images of border cell groups expressing PA-RacQ61L/PA-Cdc42T17N (**d**) or PA-RacT17N/PA-Cdc42T17N (**e**), together with LifeAct-RFP to monitor subcellular F-actin signals. Dotted blue circle labelling the PA regions with blue light illumination. PA means photo-activation. **f, g.** Time-lapse quantifications of cable discontinuity (**f**) and the ratio between cable F-actin signals and total peripheral F-actin signals (**g**) in the indicated border cell groups, before and after photo-activation of the indicated PA-Rac and PA-Cdc42 at leader border cell protrusions. **h.** Quantification of mean migration speed (μm per minute) in the indicated border cell groups, after photo-activation of the PA-Cdc42 or/and PA-Rac at leader border cell protrusions, compared with the photo-treated WT border cell groups. Scale bars are 2 μm in **a**, and 10 μm in **d** and **e**. Boxplot shows medians, 25th and 75th percentiles as box limits, minimum and maximum values as whiskers; each datapoint is displayed as a dot (from n biologically independent samples for each border cell group), in **b, c** and **h.** Data are presented as mean values +/− SD in **f** and **g** (from n biologically independent samples for each border cell group). P values by two-sided Mann–Whitney test have been listed in Supplementary Note 1. Source data are provided as a Source Data file.

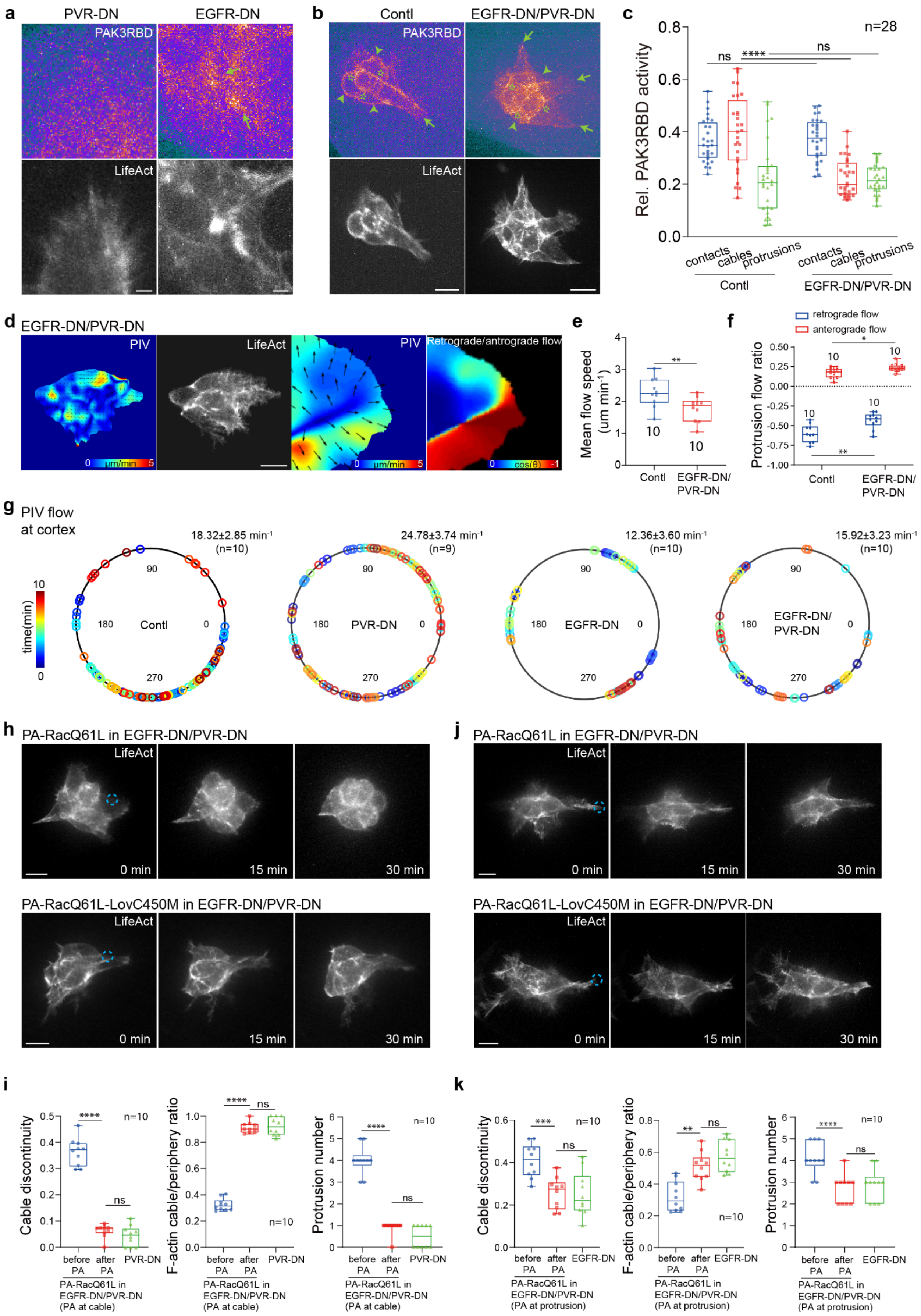

a
PVR-DN
EGFR-DN
PAK3RBD
LifeAct
b
Contl
EGFR-DN/PVR-DN
PAK3RBD
LifeAct
c
n=28
Rel. PAK3RBD activity
ns
****
ns
contacts
cables
protrusions
Contl
EGFR-DN/PVR-DN
d
EGFR-DN/PVR-DN
PIV
LifeAct
PIV
Retrograde/antrograde flow
um/min
cos(θ)
e
Mean flow speed (um min-1)
**
Contl
EGFR-DN/ PVR-DN
f
retrograde flow
anterograde flow
Protrusion flow ratio
Contl
EGFR-DN/ PVR-DN
g
PIV flow at cortex
time(min)
18.32±2.85 min-1 (n=10)
Contl
24.78±3.74 min-1 (n=9)
PVR-DN
12.36±3.60 min-1 (n=10)
EGFR-DN
15.92±3.23 min-1 (n=10)
EGFR-DN/ PVR-DN
h
PA-RacQ61L in EGFR-DN/PVR-DN
LifeAct
0 min
15 min
30 min
PA-RacQ61L-LovC450M in EGFR-DN/PVR-DN
j
PA-RacQ61L in EGFR-DN/PVR-DN
PA-RacQ61L-LovC450M in EGFR-DN/PVR-DN
i
Cable discontinuity
F-actin cable/periphery ratio
Protrusion number
n=10
before PA
after PA
PVR-DN
PA-RacQ61L in EGFR-DN/PVR-DN (PA at cable)
k
Cable discontinuity
F-actin cable/periphery ratio
Protrusion number
before PA
after PA
EGFR-DN
PA-RacQ61L in EGFR-DN/PVR-DN (PA at protrusion)

Supplementary Figure 9. PVR and EGFR guide Rac1 activity and actin flows at protrusions and cables of migrating border cells.

a. Representative PAK3RBD-GFP and F-actin images in the indicated groups for signal mis-localization. **b.** Representative PAK3RBD-GFP and F-actin images in the indicated groups. Green arrows marking protrusions, green arrowheads marking cables, while green stars marking border cell-cell contacts. **c.** Quantification of relative PAK3RBD-GFP intensity located at contacts, cables or protrusions in the indicated groups. **d.** Representative PIV analyses of actin flows in the whole group and at protrusions, and retrograde/anterograde direction analysis of actin flows at protrusions in the indicated group. **e, f, g.** Quantification of mean flow speed (μm per minute) at protrusions (**e**), the occurrence ratio of protrusion retrograde and anterograde actin flows (**f**) and actin flows occurring at cables (**g**) in the indicated border cell groups. In **g**, RGB colour marking the time window; number at perimeter showing the angle degree where actin flows occur during the indicated time window. Number above the circle showing the occurrence number of actin flows at cable with mean values +/− SD. **h, j.** Representative time-lapse F-actin images of the indicated groups, before and after photo-activation of PA-RacQ61L at cables (**h**) or protrusions (**j**) of one border cell. Dotted blue circle labelling the PA regions with blue light illumination. PA means photo-activation. **i, k.** Quantifications of cable discontinuity, the ratio between cable F-actin signals and total peripheral F-actin signals, and total cell area in the groups expressing PA-RacQ61L, PVR-DN and EGFR-DN, before and after 20-30−minute photo-activation at cables (**i**) or protrusions (**k**) of one border cell. Scale bars are 1 μm in **a**, and 10 μm in **b, d, h** and **j**. Boxplot shows medians, 25th and 75th percentiles as box limits, minimum and maximum values as whiskers; each datapoint is displayed as a dot (from n biologically independent samples for each border cell group), in **c, e, f, i** and **k**. P values by two-sided Mann–Whitney test have been listed in Supplementary Note 1. Source data are provided as a Source Data file.

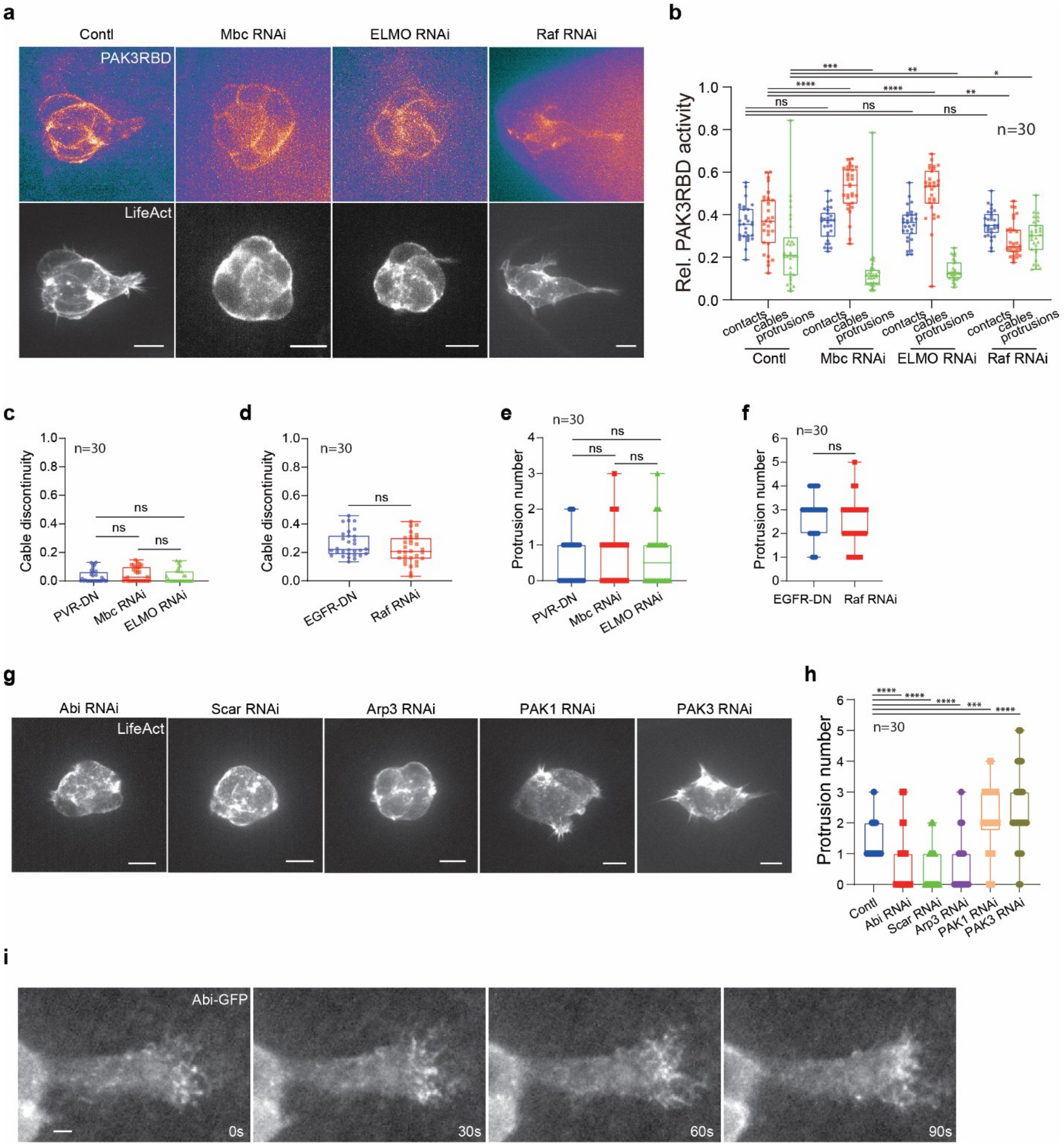

Supplementary Figure 10. Characterization of the intermediators between guidance receptors and Rac1, and of the effectors downstream of Rac1 in controlling border cell protrusions.

a. Representative PAK3RBD-GFP and F-actin images in border cell groups expressing Mbc RNAi, ELMO RNAi, Raf RNAi or control, together with LifeAct-RFP to discriminate and label different regions enriched with subcellular F-actin signals. **b.** Quantification of relative PAK3RBD-GFP intensity located at contacts, cables or protrusions in the indicated border cell groups. **c, d.** Quantification of cable discontinuity in border cell groups expressing PVR-DN, Mbc RNAi or ELMO RNAi (**c**), EGFR-DN or Raf RNAi (**d**). **e, f.** Quantification of protrusion number in border

cell groups expressing PVR-DN, Mbc RNAi or ELMO RNAi (**e**), EGFR-DN or Raf RNAi (**f**). **g.** Representative F-actin images of border cell groups expressing Abi RNAi, Scar RNAi, Arp3 RNAi, PAK1 RNAi or PAK3 RNAi, together with LifeAct-RFP to monitor subcellular F-actin signals. **h.** Quantification of protrusion number in border cell groups expressing Abi RNAi, Scar RNAi, Arp3 RNAi, PAK1 RNAi or PAK3 RNAi. **i.** Representative time-lapse images of border cell groups expressing Abi-GFP, driven by *Slbo-Gal4/UAS* genetic tool. The results of these time-lapse images for Abi-GFP distribution pattern at protrusion tips have been successfully repeated from the at least 3 independent experiments. Scale bars are 2 μm in **i**, and 10 μm in **a** and **g**. Boxplot shows medians, 25th and 75th percentiles as box limits, minimum and maximum values as whiskers; each datapoint is displayed as a dot (from n biologically independent samples for each border cell group), in **b-f, h**. P values by two-sided Mann–Whitney test have been listed in Supplementary Note 1. Source data are provided as a Source Data file.

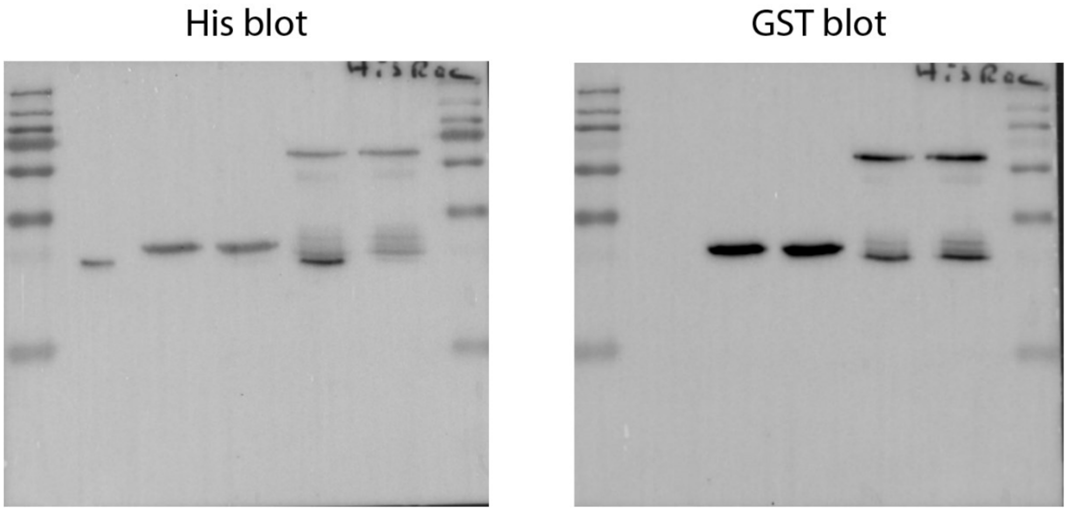

Supplementary Figure 11. Original data of western blot analyses shown in supplementary figure 2. The results have been successfully repeated from the at least 3 independent experiments.

Supplementary Note 1. Figures P value

**Figure 1. b**	
balanced *vs.* loose	<0.0001
loose *vs.* tight	<0.0001
balanced *vs.* tight	<0.0001

**Figure 1. c**	
balanced *vs.* loose	<0.0001
loose *vs.* tight	<0.0001
balanced *vs.* tight	<0.0001

**Figure 1. d**	
balanced *vs.* loose	<0.0001
loose *vs.* tight	<0.0001
balanced *vs.* tight	<0.0001

**Figure 1. e**	contacts	cables	protrusions
balanced *vs.* loose	0.04	<0.0001	<0.0001
loose *vs.* tight	0.0476	<0.0001	<0.0001
balanced *vs.* tight	0.9487	<0.0001	<0.0001

**Figure 1. f**	
balanced *vs.* loose	<0.0001
loose *vs.* tight	<0.0001
balanced *vs.* tight	<0.0001

**Figure 1. g**	cables	protrusions
front *vs.* middle	<0.0001	<0.0001
front *vs.* rear	<0.0001	<0.0001
middle *vs.* rear	<0.0001	0.0012

**Figure 1. j**	cables	protrusions
balanced *vs.* loose	<0.0001	<0.0001
loose *vs.* tight	<0.0001	<0.0001
balanced *vs.* tight	<0.0001	<0.0001

**Figure 2. b**	contacts	cables	protrusions
balanced *vs.* loose	0.2359	<0.0001	<0.0001
loose *vs.* tight	0.1462	<0.0001	<0.0001
balanced *vs.* tight	0.8545	<0.0001	<0.0001

**Figure 2. c**	balanced	loose	tight
front *vs.* middle	0.8545	0.3504	0.8545
front *vs.* rear	0.665	0.2301	0.4581
middle *vs.* rear	0.3354	0.7973	0.8545

**Figure 2. d**	cables	protrusions
front *vs.* middle	0.0003	0.0063
front *vs.* rear	0.0084	0.0105
middle *vs.* rear	0.1973	0.4986

**Figure 3. b**	PA-RacT17N (PA at leader cell cables)	PA-RacT17N (PA at leader cell protrusions)	PA-RacQ61L (PA at leader cell cables)	PA-RacQ61L (PA at leader cell protrusions)
before PA *vs.* after PA	<0.0001	<0.0001	<0.0001	0.0005

**Figure 3. c**	PA-RacT17N (PA at leader cell cables)	PA-RacT17N (PA at leader cell protrusions)	PA-RacQ61L (PA at leader cell cables)	PA-RacQ61L (PA at leader cell protrusions)
before PA *vs.* after PA	0.0005	0.0003	0.0084	0.0005

**Figure 4. d**	photo treated cell	nearby	far away
0 min *vs.* 5 min	0.0001	0.0029	0.0115
5 min *vs.* 10 min	<0.0001	0.0001	<0.0001
10 min *vs.* 15 min	<0.0001	<0.0001	<0.0001
**Figure 4. h**	photo treated cell	nearby	far away
0 min *vs.* 5 min	<0.0001	<0.0001	<0.0001
5 min *vs.* 10 min	<0.0001	<0.0001	<0.0001
10 min *vs.* 15 min	0.0029	0.1431	0.0232

**Figure 5. b**	contacts	cables	protrusions
Contl *vs.* Rho1DN	0.8137	< 0.0001	0.0002
Contl *vs.* Rho1CA	< 0.0001	< 0.0001	0.0602
**Figure 5. d**	cables	protrusions	
0 sec *vs.* 180 sec	0.0002	0.0002	
**Figure 5. f**	cables	protrusions	
0 sec *vs.* 180 sec	>0.9999	0.7209	
**Figure 5. h**	cables	protrusions	
0 min *vs.* 12 min	0.003	0.0002	
**Figure 5. J**	cables	protrusions	
0 min *vs.* 12 min	0.6454	0.8785	
**Figure 5. l**	cables	protrusions	
0 min *vs.* 12 min	0.9591	0.7984	

**Figure 6. b**		
Contl *vs.* PA-RacQ61L	0.7103	
Contl *vs.* PA-RacT17N	0.3494	
Contl *vs.* PA-Cdc42Q79L	<0.0001	
Contl *vs.* PA-Cdc42T17N	<0.0001	
**Figure 6. c**	retrograde flow	anterograde flow
Contl *vs.* PA-RacQ61L	0.4119	0.5516
Contl *vs.* PA-RacT17N	0.5573	0.6539
Contl *vs.* PA-Cdc42Q79L	0.0197	0.0127
Contl *vs.* PA-Cdc42T17N	0.0159	0.0430
**Figure 6. d**		
Contl *vs.* PA-Cdc42Q79L	<0.0001	
Contl *vs.* PA-Cdc42T17N	<0.0001	
PA-Cdc42Q79L *vs.* PA-Cdc42T17N	<0.0001	

**Figure 7. b**	contacts	cables	protrusions
Contl *vs.* PVR-DN	0.4689	< 0.0001	< 0.0001
Contl *vs.* EGFR-DN	0.5310	< 0.0001	0.0026
**Figure 7. e**			
Contl *vs.* PVR-DN	0.0029		
Contl *vs.* EGFR-DN	0.9048		
PVR-DN *vs.* EGFR-DN	0.0001		
**Figure 7. f**	retrograde flow	anterograde flow	
Contl *vs.* PVR-DN	0.0001	0.0115	
Contl *vs.* EGFR-DN	0.8421	0.4967	
**Figure 7. h**	cable discontinuity	F-actin cable/periphery ratio	protrusion number
before PA *vs.* after PA	<0.0001	<0.0001	<0.0001
**Figure 7. j**	cable discontinuity	F-actin cable/periphery ratio	protrusion number
before PA *vs.* after PA	<0.0001	<0.0001	<0.0001

Supplementary Figures P value

**Supplementary Figure 1. b**			
balanced *vs.* loose	<0.0001		
loose *vs.* tight	<0.0001		
balanced *vs.* tight	<0.0001		
**Supplementary Figure 1. c**			
balanced *vs.* loose	<0.0001		
loose *vs.* tight	<0.0001		
balanced *vs.* tight	<0.0001		
**Supplementary Figure 1. d**	balanced	loose	tight
front *vs.* middle	<0.0001	0.2301	<0.0001
front *vs.* rear	<0.0001	0.2589	0.005
middle *vs.* rear	0.2301	0.909	0.4133
**Supplementary Figure 1. f**			
balanced *vs.* loose	<0.0001		
loose *vs.* tight	<0.0001		
balanced *vs.* tight	<0.0001		

**Supplementary Figure 3. b**	contacts	cables	protrusions
Contl *vs.* Rac1DN	0.3489	<0.0001	< 0.0001
Contl *vs.* Cdc42DN	<0.0001	0.9109	< 0.0001
**Supplementary Figure 3. e**	contacts	cables	protrusions
Contl vs.Rac2 LOF	0.6228	0.786	0.9007
Contl vs. Rac3 RNAi	0.7635	0.9824	0.9007
Contl vs. Rac RNAi	0.4147	< 0.0001	0.0015
Contl vs. Cdc42 RNAi	< 0.0001	0.9124	< 0.0001
**Supplementary Figure 3. g**			
Contl vs.Rac1 RNAi	< 0.0001		
Contl vs. Cdc42 RNAi	< 0.0001		

**Supplementary Figure 4. b**	PA-RacT17N (PA at border cell-to-cell contacts)	PA-RacT17N-LovC450M (PA at leader cell cables)	PA-RacQ61L-LovC450M (PA at leader cell cables)	PA-RacT17N (PA at rear cell cables)	PA-RacQ61L (PA at rear cell cables)
before PA *vs.* after PA	0.9727	0.9099	0.9105	0.0002	0.001

**Supplementary Figure 5. a-c**	cable discontinuity	F-actin cable/periphery ratio	BC area	
before PA *vs.* after PA	0.8126	0.6987	0.7959	
**Supplementary Figure 5. d-f**	PA-RacT17N	PA-RacQ61L	PA-RacT17N-LovC450M	PA-RacQ61L-LovC450M
before PA *vs.* after PA (cable discontinuity)	<0.0001	<0.0001	0.8973	0.7959
before PA *vs.* after PA (F-actin cable/periphery ratio)	<0.0001	<0.0001	0.4665	0.7004
before PA *vs.* after PA (BC area)	0.0147	0.0001	0.9118	0.2301
**Supplementary Figure 5. g-i**	PA-RacT17N	PA-RacQ61L		
before PA *vs.* after PA (cable discontinuity)	<0.0001	0.0021		
before PA *vs.* after PA (F-actin cable/periphery ratio)	<0.0001	0.0004		
before PA *vs.* after PA (BC area)	0.0002	0.0232		

**Supplementary Figure 5. j-l**	PA-RacT17N	PA-RacQ61L
before PA *vs.* after PA (cable discontinuity)	<0.0001	0.0001
before PA *vs.* after PA (F-actin cable/periphery ratio)	0.0001	<0.0001
before PA *vs.* after PA (BC area)	0.0185	0.0232
**Supplementary Figure 5. m, n**	PA-RacT17N	PA-RacQ61L
before PA *vs.* after PA (leader cables)	0.6305	0.469
before PA *vs.* after PA (leader protrusions)	0.6305	0.5417

**Supplementary Figure 6. b**	contacts	cables	protrusions
Contl *vs.* ROCK-CA	0.0017	<0.0001	0.0809
Contl *vs.*ROCK RNAi	0.7421	<0.0001	0.0005
Contl *vs.* Sqh RNAi	0.646	<0.0001	0.0003
**Supplementary Figure 6. d**	contacts	cables	protrusions
Contl *vs.* Y27632	0.7421	< 0.0001	0.0005
**Supplementary Figure 6. f**			
0 s vs. 120 s	0.002		
**Supplementary Figure 6. h**			
0 s vs. 120 s	0.002		
**Supplementary Figure 6 j**	cables	protrusions	
0 sec *vs.* 180 sec	0.0002	0.0002	
**Supplementary Figure 6. l**	cables	protrusions	
0 min *vs.* 12 min	0.0002	0.0006	

**Supplementary Figure 7. e**	cable discontinuity	F-actin cable/periphery ratio	protrusion number
before PA *vs.* after PA	0.7109	0.25	>0.9999

**Supplementary Figure 8. b**		
PA-Cdc42Q79L *vs.* PA-Cdc42Q79L/PA-RacQ61L	0.0002	
PA-Cdc42Q79L *vs.* PA-Cdc42Q79L/PA-RacT17N	0.1457	
PA-Cdc42T17N *vs.* PA-Cdc42T17N/PA-RacQ61L	0.0435	
PA-Cdc42T17N *vs.* PA-Cdc42T17N/PA-RacT17N	0.6965	
**Supplementary Figure 8. c**	retrograde flow	anterograde flow
PA-Cdc42Q79L *vs.* PA-Cdc42Q79L/PA-RacQ61L	0.0266	0.0434
PA-Cdc42Q79L *vs.* PA-Cdc42Q79L/PA-RacT17N	0.1011	0.122
PA-Cdc42T17N *vs.* PA-Cdc42T17N/PA-RacQ61L	0.9682	0.4002
PA-Cdc42T17N *vs.* PA-Cdc42T17N/PA-RacT17N	0.9654	0.8968
**Supplementary Figure 8. f**	PA-Cdc42T17N/PA-RacQ61L	PA-Cdc42T17N/PA-RacT17N
0 min *vs.* 22.5 min	0.7959	0.9116
**Supplementary Figure 8. g**	PA-Cdc42T17N/PA-RacQ61L	PA-Cdc42T17N/PA-RacT17N
0 min *vs.* 22.5 min	0.5167	0.9265
**Supplementary Figure 8. h**		
Contl *vs.* PA-Cdc42Q79L	0.0021	
Contl *vs.* PA-Cdc42Q79L/PA-RacQ61L	0.0409	
Contl *vs.* PA-Cdc42Q79L/PA-RacT17N	< 0.0001	
Contl *vs.* PA-Cdc42T17N	< 0.0001	
Contl *vs.* PA-Cdc42T17N/PA-RacQ61L	< 0.0001	
Contl *vs.* PA-Cdc42T17N/PA-RacT17N	< 0.0001	
PA-Cdc42Q79L *vs.* PA-Cdc42Q79L/PA-RacQ61L	0.3599	

**Supplementary Figure 9. c**	contacts	cables	protrusions
Contl *vs.* PVR-DN/EGFR-DN	0.6903	< 0.0001	0.4891
**Supplementary Figure 9. e**			
Contl *vs.* PVR-DN/EGFR-DN	0.0089		
**Supplementary Figure 9. f**	retrograde flow	anterograde flow	
Contl *vs.* PVR-DN/EGFR-DN	0.0052	0.0185	
**Supplementary Figure 9. i**	cable discontinuity	F-actin cable/periphery ratio	protrusion number
before PA *vs.* after PA	< 0.0001	< 0.0001	< 0.0001
after PA *vs.* PVR-DN	0.1821	0.8676	0.1409
**Supplementary Figure 9. k**	cable discontinuity	F-actin cable/periphery ratio	protrusion number
before PA *vs.* after PA	0.0007	0.0021	0.0015
after PA *vs.* EGFR-DN	0.9705	0.1655	0.7056

**Supplementary Figure 10. b**	contacts	cables	protrusions
Contl *vs.*Mbc RNAi	0.9941	< 0.0001	0.0003
Contl *vs.*ELMO RNAi	0.9357	< 0.0001	0.0063
Contl *vs.*Raf RNAi	0.9824	0.0076	0.0225
**Supplementary Figure 10. c**	cable discontinuity		
PVR-DN *vs.*Mbc RNAi	0.3954		
PVR-DN *vs.*ELMO RNAi	0.8583		
Mbc RNAi *vs.*ELMO RNAi	0.6027		
**Supplementary Figure 10. d**	cable discontinuity		
EGFR-DN *vs.*Raf RNAi	0.1257		
**Supplementary Figure 10. e**	protrusion number		
PVR-DN *vs.*Mbc RNAi	0.4501		
PVR-DN *vs.*ELMO RNAi	0.7406		
Mbc RNAi *vs.*ELMO RNAi	0.7091		
**Supplementary Figure 10. f**	protrusion number		
EGFR-DN *vs.*Raf RNAi	0.0756		
**Supplementary Figure 10. h**	protrusion number		
Contl *vs.*Abi RNAi	<0.0001		
Contl *vs.*Scar RNAi	<0.0001		
Contl *vs.*Arp3 RNAi	<0.0001		
Contl *vs.*PAK1 RNAi	0.0003		
Contl *vs.*PAK3 RNAi	<0.0001		
