# Supplementary material for: Two Rac1 pools integrate the direction and coordination of collective cell migration
Source: Nat Commun. 2022 Oct 12;13:6014. doi: 10.1038/s41467-022-33727-6 (PMC9556596; doi:10.1038/s41467-022-33727-6)
Supplement: Supplementary file 3 — Description of Additional Supplementary Files [file 41467_2022_33727_MOESM3_ESM.pdf]

Description of Additional Supplementary Files

Supplementary Movie 1: Time-lapse images of actin flow PIV, F-actin and Myosin-II signals at the balanced migrating border cells. Scale bar is 10 μm.

Supplementary Movie 2: Time-lapse images of Rac1 photo-inhibitory effect on a border cell group expressing LifeActRFP and PA-RacT17N, after the focal inhibition of Rac at leader border cell cables. Scale bar is 10 μm. Dotted circles marking the PA regions in border cells; PA means photo-activation per 30 seconds.

Supplementary Movie 3: Time-lapse images of Rac1 photo-inhibitory effect on a border cell group expressing LifeActRFP and PA-RacT17N, after the focal inhibition of Rac at leader border cell protrusions. Scale bar is 10 μm. Dotted circles marking the PA regions in border cells; PA means photo-activation per 30 seconds.

Supplementary Movie 4: Time-lapse images of PIV, divergence and direction of actin flows at protrusions of leader border cells expressing PA-Cdc42Q61L, after the focal activation of Cdc42 at leader border cell protrusion tips. Dotted circle marking the PA region in border cell leading protrusion; PA means photo-activation per 10 seconds.

Supplementary Movie 5: Time-lapse images of PIV, divergence and direction of actin flows at protrusions of leader border cells expressing PA-Cdc42T17N, after the focal inhibition of Cdc42 at leader border cell protrusion tips. Dotted circle marking the PA region in border cell leading protrusion; PA means photo-activation per 10 seconds.

Supplementary Movie 6: Time-lapse images of PIV and direction of actin flows at protrusions of WT border cells. Arrows marking the boundary between retrograde actin flows and cable actin flows when these two flows converge with each other; while arrows vanishing when these two flows separate from each other.

Supplementary Movie 7: Time-lapse images of PIV of actin flows from border cell groups with the inhibition of PVR, EGFR or both receptors.

Supplementary Movie 8: Time-lapse images of direction of actin flows at protrusions from border cell groups with the inhibition of PVR, EGFR or both receptors.
