# Supplementary material for: Two Rac1 pools integrate the direction and coordination of collective cell migration
Source: Nat Commun. 2022 Oct 12;13:6014. doi: 10.1038/s41467-022-33727-6 (PMC9556596; doi:10.1038/s41467-022-33727-6)
Supplement: Supplementary file 12 — Reporting Summary [file 41467_2022_33727_MOESM12_ESM.pdf]

nature portfolio

Corresponding author(s): Xiaobo Wang

Last updated by author(s): Sep 26, 2022

Reporting Summary

Nature Portfolio wishes to improve the reproducibility of the work that we publish. This form provides structure for consistency and transparency in reporting. For further information on Nature Portfolio policies, see our Editorial Policies and the Editorial Policy Checklist.

Statistics

For all statistical analyses, confirm that the following items are present in the figure legend, table legend, main text, or Methods section.

n/a	Confirmed	
☐	☒	The exact sample size (*n*) for each experimental group/condition, given as a discrete number and unit of measurement
☐	☒	A statement on whether measurements were taken from distinct samples or whether the same sample was measured repeatedly
☐	☒	The statistical test(s) used AND whether they are one- or two-sided<br>*Only common tests should be described solely by name; describe more complex techniques in the Methods section.*
☐	☒	A description of all covariates tested
☒	☐	A description of any assumptions or corrections, such as tests of normality and adjustment for multiple comparisons
☐	☒	A full description of the statistical parameters including central tendency (e.g. means) or other basic estimates (e.g. regression coefficient) AND variation (e.g. standard deviation) or associated estimates of uncertainty (e.g. confidence intervals)
☐	☒	For null hypothesis testing, the test statistic (e.g. F, t, r) with confidence intervals, effect sizes, degrees of freedom and P value noted<br>*Give P values as exact values whenever suitable.*
☒	☐	For Bayesian analysis, information on the choice of priors and Markov chain Monte Carlo settings
☒	☐	For hierarchical and complex designs, identification of the appropriate level for tests and full reporting of outcomes
☒	☐	Estimates of effect sizes (e.g. Cohen's *d*, Pearson's *r*), indicating how they were calculated

Our web collection on statistics for biologists contains articles on many of the points above.

Software and code

Policy information about availability of computer code

Data collection	Imaging data have been collected by Leica Metamorph software (version: Metamorph 7.8.13.0).
Data analysis	GraphPad Prism software (version: 8.0.2) has been used for box and whiskers plots. ImageJ software (version: 1.53f51) has been used for quantifications. MATLAB software (version: R2020b) has been used for photo-bleaching, correction and nuclear centers tracking, the particle image velocimetry (PIV) analysis (actin flow codes run by Matlab) and also identification of cell edge feature (CellGeo codes run by Matlab).

For manuscripts utilizing custom algorithms or software that are central to the research but not yet described in published literature, software must be made available to editors and reviewers. We strongly encourage code deposition in a community repository (e.g. GitHub). See the Nature Portfolio guidelines for submitting code & software for further information.

Data

Policy information about availability of data

All manuscripts must include a data availability statement. This statement should provide the following information, where applicable:

- Accession codes, unique identifiers, or web links for publicly available datasets
- A description of any restrictions on data availability
- For clinical datasets or third party data, please ensure that the statement adheres to our policy

The source data underlying Figs 1b-g,i,j, 2b-d, 3b-g, 4b-d,f-h, 5b, d, f, h-l, 6b-d, g, 7b,e,f,h and j, Supplementary Figs 1b-d, f-h, 3b, e, g, 4b, 5a-o, 6b, d, f, h, j, 7e, 8b, c, f-h, 9c, e-g, i, k 10b-f and h are provided as a Source Data file.
Complete data are available in the main article, supplementary materials, and source data files. Since all the raw confocal imaging data supporting the findings of this study runs more than two terabytes and in multiple files, we have not submitted it to the public repository but preserved in our NAS drive and are freely

available from the corresponding author (Contact Address: xiaobo.wang@univ-tlse3.fr). Representative images are in the main or supplementary figures. Source data are provided with this paper.

Field-specific reporting

Please select the one below that is the best fit for your research. If you are not sure, read the appropriate sections before making your selection.

- [x] Life sciences
- [ ] Behavioural & social sciences
- [ ] Ecological, evolutionary & environmental sciences

For a reference copy of the document with all sections, see nature.com/documents/nr-reporting-summary-flat.pdf

Life sciences study design

All studies must disclose on these points even when the disclosure is negative.

Sample size	The experiments were performed, in general, on the 28-120 border cell groups for PAK3RBD-GFP and F-actin signals, and 8-13 independent samples for optogenetics and PIV analyses. Collection of PAK3RBD-GFP and F-actin images are very convenient so that we can collect more than 28 border cell samples for better statistical quantification (normally more than 25 samples are sufficient to compare the phenotypes for border cells). However, optogenetics and actin flows by imaging are much more difficult, so that we chose at least 8 experimental samples for phenotype comparison. Choice of all samples is unbiased.<br>The exact number of analyzed samples is specified for each experiment in the corresponding figure and/or figure legends.
Data exclusions	No data were excluded from the analysis.
Replication	The experiments were replicated or performed independently at least 3 times, and the exact number of independent experiment is listed in the corresponding figure legends.
Randomization	Sample allocation was random.
Blinding	We were blinded to group allocation during data collection and analysis.

Reporting for specific materials, systems and methods

We require information from authors about some types of materials, experimental systems and methods used in many studies. Here, indicate whether each material, system or method listed is relevant to your study. If you are not sure if a list item applies to your research, read the appropriate section before selecting a response.

Materials & experimental systems

n/a	Involved in the study	
[ ]	[x]	Antibodies
[x]	[ ]	Eukaryotic cell lines
[x]	[ ]	Palaeontology and archaeology
[ ]	[x]	Animals and other organisms
[x]	[ ]	Human research participants
[x]	[ ]	Clinical data
[x]	[ ]	Dual use research of concern

Methods

n/a	Involved in the study	
[x]	[ ]	ChIP-seq
[x]	[ ]	Flow cytometry
[x]	[ ]	MRI-based neuroimaging

Antibodies

Antibodies used	His antibody (Invitrogen, Clone name: HIS.H8; Catalogue number: MA1-21315; 1:1000 dilution); GST antibody (Invitrogen, Clone name: 8-326; Catalogue number: MA4-004; 1:1000 dilution).
Validation	Validation information of each primary antibody for the species and application is available at the following manufacturer's websites or our previous publication:<br>https://www.thermofisher.com/antibody/product/6x-His-Tag-Antibody-clone-HIS-H8-Monoclonal/MA1-21315<br>https://www.thermofisher.com/antibody/product/GST-Tag-Antibody-clone-8-326-Monoclonal/MA4-004

Animals and other organisms

Policy information about studies involving animals; ARRIVE guidelines recommended for reporting animal research

Laboratory animals	Drosophila melanogaster, both male and female, 3-5 days after adult flies are born. Specific strains are as follows: Sqh::RLCmyosinII–mCherry (from Eric E. Wieschaus), Slbo-Gal4 (from Pernille Rorth), UAS-Abi-GFP (from Sven Bogdan), slbo::LifeAct–GFP (from this

study), slbo::LifeAct–RFP (from this study), UASt-PA-RacQ61L (from this study), UASt-PA-RacT17N (from this study), UASt-PA-RacQ61L-LovC450M (from this study), UASt-PA-RacT17N-LovC450M (from this study), UASt-PA-Cdc42Q61L (from this study), UASt-PA-Cdc42T17N (from this study), UASp-CIBN-CAAX/UASp-Cry2-RhoGEF2 (Opto-RhoGEF tool from Stefano De Renzis), UASp-CIBN-CAAX/UASp-Cry2-Rho1DN (Opto-Rho1DN tool from Bing He), and all these following stocks are from Bloomington Drosophila stock center: UAS-Rac1DN (BL6292), UAS-Cdc42DN (BL6288), UAS-Rho1CA (BL7330), UAS-Rho1DN (BL7327), UAS-ROCKCA (BL6668), UAS-ROCKRNAi (BL34324), UAS-SqhRNA (BL34939), UAS-Rac1RNAi (BL34910), Rac2Δ ry506 (BL6675), UAS-Rac3RNAi (BL51932), UAS-Cdc42RNAi (BL35756),UAS-ShgRNAi (BL32904), UAS-MbcRNAi (BL51460), UAS-ELMORNAi (BL28556), UAS-RafRNAi (BL55863), UAS-ScarRNAi (BL51803), UAS-AbiRNAi (BL51455), UAS-Arp3RNAi (BL32921), UAS-PAK1RNAi (BL28945), UAS-PAK3RNAi (BL42664), Rac1-GFP (BL52284), Rac2-GFP (BL52286), Rac3-GFP (BL37970), Cdc42-RFP (BL42236) and Sqh::PAK3-RBD-GFP (BL 52303 and BL52304 combined together).

Wild animals	No wild animals were used in the study.
Field-collected samples	No field collected samples were used in the study.
Ethics oversight	Ethical approval was not required for this study

Note that full information on the approval of the study protocol must also be provided in the manuscript.
